# Longitudinal analysis of pre‐ and post‐treatment measurements with equal baseline assumptions in randomized trials

**DOI:** 10.1002/bimj.201800389

**Published:** 2019-08-08

**Authors:** Ikuko Funatogawa, Takashi Funatogawa

**Affiliations:** ^1^ Department of Statistical Data Science The Institute of Statistical Mathematics Tachikawa Tokyo Japan; ^2^ Clinical Science and Strategy Department Chugai Pharmaceutical Co., Ltd. Chuo‐ku Tokyo Japan

**Keywords:** analysis of covariance, longitudinal analysis, pretest, posttest, repeated measure, unequal variance

## Abstract

For continuous variables of randomized controlled trials, recently, longitudinal analysis of pre‐ and posttreatment measurements as bivariate responses is one of analytical methods to compare two treatment groups. Under random allocation, means and variances of pretreatment measurements are expected to be equal between groups, but covariances and posttreatment variances are not. Under random allocation with unequal covariances and posttreatment variances, we compared asymptotic variances of the treatment effect estimators in three longitudinal models. The data‐generating model has equal baseline means and variances, and unequal covariances and posttreatment variances. The model with equal baseline means and unequal variance–covariance matrices has a redundant parameter. In large sample sizes, these two models keep a nominal type I error rate and have high efficiency. The model with equal baseline means and equal variance–covariance matrices wrongly assumes equal covariances and posttreatment variances. Only under equal sample sizes, this model keeps a nominal type I error rate. This model has the same high efficiency with the data‐generating model under equal sample sizes. In conclusion, longitudinal analysis with equal baseline means performed well in large sample sizes. We also compared asymptotic properties of longitudinal models with those of the analysis of covariance (ANCOVA) and *t*‐test.

## 1 INTRODUCTION

When primary endpoints of randomized controlled trials are continuous variables, analysis of covariance (ANCOVA) on posttreatment measurements with pretreatment measurements as a covariate is often used to compare two treatment groups. More recently, longitudinal analysis of pre‐ and posttreatment measurements as bivariate responses is also one of analytical methods to compare two treatment groups.

Under random allocation, means and variances of pretreatment measurements are expected to be equal between groups. However, unequal covariances of pre‐ and posttreatment measurements and unequal variances of posttreatment measurements between groups are often observed in clinical trials, particularly in placebo‐controlled trials. This variance–covariance structure has been discussed (Yang & Tsiatis, 2001). Chen (2006) considered the variance–covariance structure with unequal variance–covariance matrices including pretreatment variances in simulation studies. Winkens, van Breukelen, Schouten, and Berger (2007) also examined the structure with equal pretreatment variances and unequal covariances and posttreatment variances analytically, but they used the completely heterogeneous covariance matrices in their data analysis. Both Chen (2006) and Winkens et al. (2007) commented on the efficiency loss caused by not assuming equal pretreatment variances. The variance–covariance structure with equal variance–covariance matrices is also used (Chen, 2006; Winkens et al., 2007). The differences in asymptotic properties between the models with and without the constraints of equal baseline assumptions remain unclear. We can consider several longitudinal models based on the assumptions on pretreatment means and elements of variance–covariance matrices. Liang and Zeger (2000) used the model with equal pretreatment means under random allocation, and we also use this mean structure. Chen (2006), Winkens et al. (2007), and Yang and Tsiatis (2001) studied longitudinal analysis of pre‐ and posttreatment measurements in randomized trials and compared them with ANCOVAs or *t*‐tests. However, detailed comparisons among various models have not been conducted.

We compare the properties of models. The ANCOVA has higher power to detect a treatment effect than the *t*‐test under standard assumptions. In previous studies, we examined the asymptotic properties of four ANCOVA models under the cases that covariances and posttreatment variances differ between groups (Funatogawa & Funatogawa, 2011; Funatogawa, Funatogawa, & Shyr, 2011). The actual type I error rate of the usual ANCOVA with equal slopes (coefficients of pretreatment measurements) and equal residual variances is asymptotically at a nominal level only under equal sample sizes, but that of the ANCOVA with equal slopes and unequal residual variances is asymptotically at a nominal level even under unequal sample sizes (Funatogawa et al., 2011). In unequal sample sizes, an assumption of unequal residual variances is important. However, the efficiency of the latter model is relatively low. The ANCOVA with unequal slopes has higher efficiency but cannot keep a nominal type I error rate irrespective of variance assumptions (Funatogawa & Funatogawa, 2011). Yang and Tsiatis (2001) showed the longitudinal model with equal baseline means and variances and unequal covariances and posttreatment variances has the same efficiency with the ANCOVA with unequal slopes. It would be beneficial to determine whether there are methods which have a high efficiency and keep a nominal type I error rate.

In this paper, we consider three longitudinal models and compare these with four ANCOVAs and the *t*‐test on change and the *t*‐test on posttreatment measurements. We investigate whether these models asymptotically keep a nominal type I error rate and the order of efficiency analytically based on the asymptotic variances of the treatment effect estimators and the model‐based asymptotic variances. We also conduct simulation studies using the Kenward–Roger approximation (Kenward & Roger, 1997) for longitudinal models. The structure of this paper is as follows. In Section 2, we show a motivating example. In Section 3, we show the asymptotic properties of longitudinal models as well as the ANCOVAs and *t*‐tests. In Section 4, we compare longitudinal models with the ANCOVAs and *t*‐tests through simulation studies and actual data analysis. In Section 5, we offer a conclusion and discussion.

## 2 A MOTIVATING EXAMPLE

**Figure 1:**
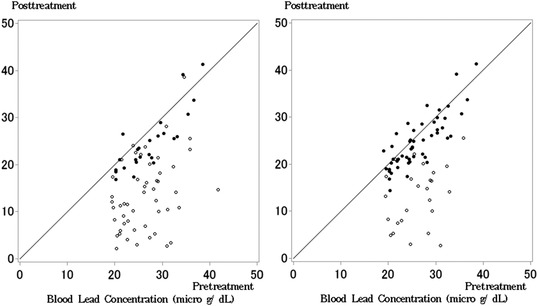
Blood lead levels at baseline and one week after administration of succimer and placebo. The open and closed circles show the succimer and placebo groups, respectively. Left panel shows all succimer data and half of the placebo data from a subsample of 100 children. Right panel shows half of the succimer data and all placebo data

As an actual example of unequal covariances and posttreatment variances between groups, we show the results of a placebo‐controlled, randomized trial of succimer in children (Rogan et al., 2001). The blood lead levels of a subsample of 100 children are analyzed in Fitzmaurice, Laird, and Ware (2004). The sample sizes are equal between two groups. Funatogawa et al. (2011) and Funatogawa and Funatogawa (2011) used these data at baseline and one week after administration as an example of unequal covariances and posttreatment variances in order to show the properties of ANCOVAs under random allocation. In this paper, we added small random values to provide the data. Because unequal sample sizes affect the results, we produced the data with unequal sample sizes randomly reducing half of data from one group, as shown in Figure 1. The left panel shows all succimer data and half of the placebo data, and the right panel shows half of the succimer data and all placebo data. We analyze these data in Section 4.

The means and variances of the pretreatment measurements were almost the same between groups because of random allocation, whereas the covariances and the means and variances of the posttreatment measurements obviously differed. The data show that changes after the intervention were small in the placebo group, and the correlation between the pre‐ and posttreatment measurements was relatively strong. The changes after the intervention were large in the succimer group, and the amounts of change differed among individuals. Then, the correlation of pre‐ and posttreatment measurements was weak. The variance of posttreatment measurements was larger than that of the placebo group, and also than that of pretreatment measurements.

## 3 LONGITUDINAL MODELS FOR PRE‐ AND POSTTREATMENT MEASUREMENTS

### 3.1 Data‐generating model

We describe a data‐generating model assuming only random allocation. We consider a randomized trial of two treatment groups. Let $Y_{ijk}$ denote, for the *i*‐th (i=1,2) treatment group, the measurement of the *j*‐th $\left(j=1,\ldots ,n_{i}\right)$ subject at the *k*‐th (k=1,2) time. $Y_{ij1}$ and $Y_{ij2}$ are pre‐ and posttreatment measurements, respectively. We assume that the pair ($Y_{ij1}$ and $Y_{ij2}$) has finite second moments and is distributed with the following mean and variance–covariance matrix:

$$\mathrm{Mean}\left(Y_{ij1},Y_{ij2}\right)=\left(\begin{array}{c} \mu_{\mathrm{pre}}\\ \mu_{i\;\mathrm{post}} \end{array}\right),\;\mathrm{Cov}\left(Y_{ij1},Y_{ij2}\right)=\left(\begin{array}{cc} \sigma_{\mathrm{pre}}^{2} & \sigma_{i\;\mathrm{cov}}\\ \sigma_{i\;\mathrm{cov}} & \sigma_{i\;\mathrm{post}}^{2} \end{array}\right).$$

Random allocation guarantees equal values for μ_pre_ and $\sigma_{\mathrm{pre}}^{2}$ between groups, but not for $\mu_{i\;\mathrm{post}}$, $\sigma_{i\;\mathrm{cov}}$, or $\sigma_{i\;\mathrm{post}}^{2}$. Because μ_pre_ is equal between groups, $\mu_{2\mathrm{post}}-\mu_{1\mathrm{post}}$ can be considered as the treatment effect. Here, we do not assume normality.

### 3.2 Longitudinal models and asymptotic properties

We consider three longitudinal models for pre‐ and posttreatment measurements under random allocation in this section and four ANCOVAs additionally in the next section for comparison. Table 1 shows the summary of these models. The first longitudinal model is the data‐generating model, and we abbreviate it EMVUV from Equal baseline Means and Variances and Unequal covariances and posttreatment Variances. The model is

$$\begin{array}{c} \;Y_{ij}=X_{ij}\;\beta +\varepsilon_{ij}, \end{array}$$

where

$$\;Y_{ij}=\left(\begin{array}{c} Y_{ij1}\\ Y_{ij2} \end{array}\right),\;\;X_{ij}=\left(\begin{array}{ccc} 1 & 0 & 0\\ 1 & 1 & x_{ij} \end{array}\right),\;\beta =\left(\begin{array}{c} \beta_{\mathrm{int}}\\ \beta_{t}\\ \beta_{\mathrm{trt}} \end{array}\right),\;\;\varepsilon_{ij}=\left(\begin{array}{c} \varepsilon_{ij1}\\ \varepsilon_{ij2} \end{array}\right),\;\;\mathrm{Cov}\left(\varepsilon_{ij}\right)=\left(\begin{array}{cc} \sigma_{\mathrm{pre}}^{2} & \sigma_{i\;\mathrm{cov}}\\ \sigma_{i\;\mathrm{cov}} & \sigma_{i\;\mathrm{post}}^{2} \end{array}\right),$$

$\;x_{ij}=0$ for i=1, and $x_{ij}=1$ for i=2. $\varepsilon_{ijk}$ is a random error. Here, $\beta_{\mathrm{int}}=\mu_{\mathrm{pre}}$, $\beta_{\mathrm{int}}+\;\beta_{t}=\mu_{1\mathrm{post}}$, and $\beta_{\mathrm{int}}+\beta_{t}+\;\beta_{\mathrm{trt}}=\mu_{2\mathrm{post}}$. β_t_ is the time effect in the first group and $\beta_{t}+\beta_{\mathrm{trt}}$ is the time effect in the second group. We are interested in $\beta_{\mathrm{trt}}=\mu_{2\mathrm{post}}-\mu_{1\mathrm{post}}$. It is the difference in the posttreatment means between groups.

**Table 1:** Summary of longitudinal models and ANCOVAs

	Methods		Mean structures^a^	$\mathrm{Cov}(\varepsilon_{ij})$ a
Longitudinal	EMVUV: Equal baseline means and variances and unequal covariances and posttreatment variances	Correct Data‐generating model	$\mu_{\mathrm{pre}},\;\mu_{i\;\mathrm{post}}$	$\left(\begin{array}{cc} \sigma_{\mathrm{pre}}^{2} & \sigma_{i\;\mathrm{cov}}\\ \sigma_{i\;\mathrm{cov}} & \sigma_{i\;\mathrm{post}}^{2} \end{array}\right)$
	EMUV: Equal baseline means and unequal variance matrices	Redundant $\sigma_{i\;\mathrm{pre}}^{2}$	$\mu_{\mathrm{pre}},\;\mu_{i\;\mathrm{post}}$	$\left(\begin{array}{cc} \sigma_{i\;\mathrm{pre}}^{2} & \sigma_{i\;\mathrm{cov}}\\ \sigma_{i\;\mathrm{cov}} & \sigma_{i\;\mathrm{post}}^{2} \end{array}\right)$
	EMEV: Equal baseline means and equal variance matrices	Constraint σ_cov_ and $\sigma_{\mathrm{post}}^{2}$	$\mu_{\mathrm{pre}},\;\mu_{i\;\mathrm{post}}$	$\left(\begin{array}{cc} \sigma_{\mathrm{pre}}^{2} & \sigma_{\mathrm{cov}}\\ \sigma_{\mathrm{cov}} & \sigma_{\mathrm{post}}^{2} \end{array}\right)$
ANCOVA	USUV: Unequal slopes and unequal residual variances	–	$\beta_{i\;\mathrm{int}},\;\beta_{i\;\mathrm{slope}}$	$\sigma_{i\;\mathrm{res}}^{2}$
	USEV: Unequal slopes and equal residual variances	Constraint $\sigma_{\mathrm{res}}^{2}$	$\beta_{i\;\mathrm{int}},\;\beta_{i\;\mathrm{slope}}$	$\sigma_{\mathrm{res}}^{2}$
	ESUV: Equal slopes and unequal residual variances	Constraint β_slope_	$\beta_{i\;\mathrm{int}},\;\beta_{\mathrm{slope}}$	$\sigma_{i\;\mathrm{res}}^{2}$
	ESEV: Equal slopes and equal residual variances	Constraint β_slope_ and $\sigma_{\mathrm{res}}^{2}$	$\beta_{i\;\mathrm{int}},\;\beta_{\mathrm{slope}}$	$\sigma_{\mathrm{res}}^{2}$

a Redundant parameters or constraints are presented in bold.

In EMUV, Equal baseline Means and Unequal Variance matrices, the variance–covariance matrices differ between groups. This model has a redundant parameter, because $\sigma_{1\mathrm{pre}}^{2}$ and $\sigma_{2\mathrm{pre}}^{2}$ are used instead of common $\sigma_{\mathrm{pre}}^{2}$. In EMEV, Equal baseline Means and Equal Variance matrices, the variance–covariance matrices are common between groups. This model is incorrect under the situation that the covariances and posttreatment variances are different between groups, because σ_cov_ and $\sigma_{\mathrm{post}}^{2}$ are used instead of σ_1cov_, $\;\sigma_{2\mathrm{cov}}$, $\sigma_{1\mathrm{post}}^{2}$, and $\sigma_{2\mathrm{post}}^{2}$.

For each model, the maximum likelihood estimators of β are obtained by $\hat{\beta }={\left(X^{\prime }V^{-1}X\right)}^{-1}\;X^{\prime }V^{-1}Y$. Here, $Y={\left(Y_{11}^{\prime },\ldots ,Y_{1n_{1}}^{\prime },Y_{21}^{\prime },\ldots ,Y_{2n_{2}}^{\prime }\right)}^{\prime }$ and $X={\left(X_{11}^{\prime },\ldots ,X_{1n_{1}}^{\prime },X_{21}^{\prime },\ldots ,X_{2n_{2}}^{\prime }\right)}^{\prime }$. **V** is a $2\left(n_{1}+n_{2}\right)\times 2\left(n_{1}+n_{2}\right)$ block diagonal matrix of $V=\mathrm{diag}(V_{11},\ldots ,V_{1n_{1}},V_{21},\ldots ,V_{2n_{2}})$ with $V_{ij}=\mathrm{Cov}\left(\varepsilon_{ij}\right)$. The model‐based asymptotic variance of ${\hat{\beta }}_{\mathrm{trt}}$ is given by the corresponding element of $\mathrm{Cov}\;\left(\hat{\beta }\right)={\left(X^{\prime }V^{-1}X\right)}^{-1}$.

We provide the asymptotic variances of ${\hat{\beta }}_{\mathrm{trt}}$ for the three longitudinal models. Table 2 summarizes the asymptotic variances. Here and throughout, we neglect o(1/N) terms with $N=n_{1}+n_{2}$. For EMVUV, the asymptotic variance of ${\hat{\beta }}_{\mathrm{trt}}$ is given as formula (I) in Table 2. EMUV has a redundant parameter. The asymptotic variance of ${\hat{\beta }}_{\mathrm{trt}}$ is

$$\left(\frac{1}{n_{1}}+\frac{1}{n_{2}}\right)\left\{\left(\frac{n_{2}\sigma_{1\mathrm{post}}^{2}+n_{1}\sigma_{2\mathrm{post}}^{2}}{n_{1}+n_{2}}\right)-{\left(\frac{n_{1}\sigma_{1\mathrm{pre}}^{2}+n_{2}\sigma_{2\mathrm{pre}}^{2}}{n_{1}+n_{2}}\right)}^{-1}{\left(\frac{n_{2}\sigma_{1\mathrm{cov}}+n_{1}\sigma_{2\mathrm{cov}}}{n_{1}+n_{2}}\right)}^{2}\right\},$$

and it is the same as formula (I) under random allocation with $\sigma_{1\mathrm{pre}}^{2}=\sigma_{2\mathrm{pre}}^{2}$.

**Table 2:** Asymptotic variances of the treatment effect estimators for the longitudinal models, ANCOVAs, and *t*‐tests under random allocation in the cases of (A) arbitrary allocation ratios and (B) equal sample sizes

Methods	Asymptotic variances of ${\hat{\beta }}_{\mathrm{trt}}$
(A) Arbitrary *n*_1_, *n*_2_	
EMVUV EMUV ANCOVA_US^a^	$\left(\frac{1}{n_{1}}+\frac{1}{n_{2}}\right)\left\{\left(\frac{n_{2}\sigma_{1\mathrm{post}}^{2}+n_{1}\sigma_{2\mathrm{post}}^{2}}{n_{1}+n_{2}}\right)-\frac{1}{\sigma_{\mathrm{pre}}^{2}}{\left(\frac{n_{2}\sigma_{1\mathrm{cov}}+n_{1}\sigma_{2\mathrm{cov}}}{n_{1}+n_{2}}\right)}^{2}\right\}\;\left(I\right)$
EMEV ANCOVA_ESEV	$\left(\frac{1}{n_{1}}+\frac{1}{n_{2}}\right)\left\{\left(\frac{n_{2}\sigma_{1\mathrm{post}}^{2}+n_{1}\sigma_{2\mathrm{post}}^{2}}{n_{1}+n_{2}}\right)-\frac{1}{\sigma_{\mathrm{pre}}^{2}}\left(\frac{n_{1}\sigma_{1\mathrm{cov}}+n_{2}\sigma_{2\mathrm{cov}}}{n_{1}+n_{2}}\right)\left(\frac{p\sigma_{1\mathrm{cov}}+q\sigma_{2\mathrm{cov}}}{p+q}\right)\right\}\;\left(\mathrm{II}\right)$ where $p=2n_{2}-n_{1}$ and $q=2n_{1}-n_{2}$.
ANCOVA_ESUV	$\left(\frac{1}{n_{1}}+\frac{1}{n_{2}}\right)\left\{\left(\frac{n_{2}\sigma_{1\mathrm{post}}^{2}+n_{1}\sigma_{2\mathrm{post}}^{2}}{n_{1}+n_{2}}\right)-\frac{1}{\sigma_{\mathrm{pre}}^{2}}\left(\frac{n_{1}^{*}\sigma_{1\mathrm{cov}}+n_{2}^{*}\sigma_{2\mathrm{cov}}}{n_{1}^{*}+n_{2}^{*}}\right)\left(\frac{p^{*}\sigma_{1\mathrm{cov}}+q^{*}\sigma_{2\mathrm{cov}}}{p^{*}+q^{*}}\right)\right\}\;\left(\mathrm{III}\right)$ where $n_{1}^{*}=n_{1}/\sigma_{1\mathrm{res}}^{2}$, $n_{2}^{*}=n_{2}/\sigma_{2\mathrm{res}}^{2}$, $p^{*}=2n_{2}-n_{1}^{*}\left(n_{1}+n_{2}\right)/\left(n_{1}^{*}+n_{2}^{*}\right),$ and $q^{*}=2n_{1}-n_{2}^{*}\left(n_{1}+n_{2}\right)/\left(n_{1}^{*}+n_{2}^{*}\right)$.
*t*‐test_Change^a^	$\left(\frac{1}{n_{1}}+\frac{1}{n_{2}}\right)\left\{\left(\frac{n_{2}\sigma_{1\mathrm{post}}^{2}+n_{1}\sigma_{2\mathrm{post}}^{2}}{n_{1}+n_{2}}\right)+\sigma_{\mathrm{pre}}^{2}-2\left(\frac{n_{2}\sigma_{1\mathrm{cov}}+n_{1}\sigma_{2\mathrm{cov}}}{n_{1}+n_{2}}\right)\right\}\;\left(\mathrm{IV}\right)$
*t*‐test_Post^a^	$\left(\frac{1}{n_{1}}+\frac{1}{n_{2}}\right)\left(\frac{n_{2}\sigma_{1\mathrm{post}}^{2}+n_{1}\sigma_{2\mathrm{post}}^{2}}{n_{1}+n_{2}}\right)\;\left(V\right)$
(B) Under $n_{1}=n_{2}=n$	
EMVUV EMUV EMEV ANCOVA_US^a^ ANCOVA_ESEV	$\frac{2}{n}\left\{\left(\frac{\sigma_{1\mathrm{post}}^{2}+\sigma_{2\mathrm{post}}^{2}}{2}\right)-\frac{1}{\sigma_{\mathrm{pre}}^{2}}{\left(\frac{\sigma_{1\mathrm{cov}}+\sigma_{2\mathrm{cov}}}{2}\right)}^{2}\right\}\;\left(\mathrm{VI}\right)$
ANCOVA_ESUV	$\frac{2}{n}\left\{\left(\frac{\sigma_{1\mathrm{post}}^{2}+\sigma_{2\mathrm{post}}^{2}}{2}\right)-\frac{1}{\sigma_{\mathrm{pre}}^{2}}\left(\frac{\sigma_{2\mathrm{res}}^{2}\sigma_{1\mathrm{cov}}+\sigma_{1\mathrm{res}}^{2}\sigma_{2\mathrm{cov}}}{\sigma_{1\mathrm{res}}^{2}+\sigma_{2\mathrm{res}}^{2}}\right)\left(\frac{\sigma_{1\mathrm{res}}^{2}\sigma_{1\mathrm{cov}}+\sigma_{2\mathrm{res}}^{2}\sigma_{2\mathrm{cov}}}{\sigma_{1\mathrm{res}}^{2}+\sigma_{2\mathrm{res}}^{2}}\right)\right\}\;\left(\mathrm{VII}\right)$
*t*‐test_Change^a^	$\frac{2}{n}\left\{\left(\frac{\sigma_{1\mathrm{post}}^{2}+\sigma_{2\mathrm{post}}^{2}}{2}\right)+\sigma_{\mathrm{pre}}^{2}-2\left(\frac{\sigma_{1\mathrm{cov}}+\sigma_{2\mathrm{cov}}}{2}\right)\right\}\;\left(\mathrm{VIII}\right)$
*t*‐test_Post^a^	$\frac{2}{n}\left(\frac{\sigma_{1\mathrm{post}}^{2}+\sigma_{2\mathrm{post}}^{2}}{2}\right)\;\left(\mathrm{IX}\right)$

a The estimates with equal and unequal variances are identical for ANCOVAs with unequal slopes and *t*‐tests.

For EMEV, the assumed model is incorrect. The asymptotic variance of ${\hat{\beta }}_{\mathrm{trt}}$ under random allocation is the 3,3‐th element of $E[{\left(X^{\prime }V^{-1}X\right)}^{-1}\left\{\left(X^{\prime }V^{-1}\mathrm{Var}\left(Y\right)V^{-1}X\right)\right\}{\left(X^{\prime }V^{-1}X\right)}^{-1}]$, and it is given as formula (II) in Table 2. However, the model‐based asymptotic variance of ${\hat{\beta }}_{\mathrm{trt}}$ under random allocation is the 3,3‐th element of $E[{\left(X^{\prime }V^{-1}X\right)}^{-1}]$. Table 3 shows model‐based asymptotic variances which are biased from asymptotic variances given in Table 2, and the bias, model‐based asymptotic variance minus asymptotic variance. For EMEV, there are both conservative and liberal cases under unequal sample sizes, and there is no bias under equal sample sizes. Under equal sample sizes, the asymptotic variances of EMVUV, EMUV, and EMEV are the same and these are given as formula (VI) in Table 2.

**Table 3:** Biased model‐based asymptotic variances of the treatment effect estimators, bias, and bias under equal sample sizes

Methods	Model‐based asymptotic variances of ${\hat{\beta }}_{\mathrm{trt}}$, Bias^a^, Bias under *n*_1_=$\;n_{2}$
EMEV ANCOVA_ESEV	$\left(\frac{1}{n_{1}}+\frac{1}{n_{2}}\right)\left\{\left(\frac{n_{1}\sigma_{1\mathrm{post}}^{2}+n_{2}\sigma_{2\mathrm{post}}^{2}}{n_{1}+n_{2}}\right)-\frac{1}{\sigma_{\mathrm{pre}}^{2}}{\left(\frac{n_{1}\sigma_{1\mathrm{cov}}+n_{2}\sigma_{2\mathrm{cov}}}{n_{1}+n_{2}}\right)}^{2}\right\},\;$ $\frac{\left(n_{1}-n_{2}\right)(\sigma_{1\mathrm{post}}^{2}-\sigma_{2\mathrm{post}}^{2})}{n_{1}n_{2}}-\frac{2\left(n_{1}-n_{2}\right)\left(n_{1}\sigma_{1\mathrm{cov}}+n_{2}\sigma_{2\mathrm{cov}}\right)\left(\sigma_{1\mathrm{cov}}-\sigma_{2\mathrm{cov}}\right)}{n_{1}n_{2}\left(n_{1}+n_{2}\right)\sigma_{\mathrm{pre}}^{2}},\;0$
ANCOVA_USEV	$\left(\frac{1}{n_{1}}+\frac{1}{n_{2}}\right)\left\{\left(\frac{n_{1}\sigma_{1\mathrm{post}}^{2}+n_{2}\sigma_{2\mathrm{post}}^{2}}{n_{1}+n_{2}}\right)-\frac{1}{\sigma_{\mathrm{pre}}^{2}}\left(\frac{n_{1}\sigma_{1\mathrm{cov}}^{2}+n_{2}\sigma_{2\mathrm{cov}}^{2}}{n_{1}+n_{2}}\right)\right\},\;$ $\frac{\left(n_{1}-n_{2}\right)(\sigma_{1\mathrm{post}}^{2}-\sigma_{2\mathrm{post}}^{2})}{n_{1}n_{2}}-\frac{\left(n_{1}-n_{2}\right)\left(\sigma_{1\mathrm{cov}}^{2}-\sigma_{2\mathrm{cov}}^{2}\right)}{n_{1}n_{2}\sigma_{\mathrm{pre}}^{2}}-\frac{{\left(\sigma_{1\mathrm{cov}}-\sigma_{2\mathrm{cov}}\right)}^{2}}{\left(n_{1}+n_{2}\right)\sigma_{\mathrm{pre}}^{2}},\;$ $\frac{{\left(\sigma_{1\mathrm{cov}}-\sigma_{2\mathrm{cov}}\right)}^{2}}{\left(n_{1}+n_{2}\right)\sigma_{\mathrm{pre}}^{2}}$
ANCOVA_USUV	$\left(\frac{1}{n_{1}}+\frac{1}{n_{2}}\right)\left\{\left(\frac{n_{2}\sigma_{1\mathrm{post}}^{2}+n_{1}\sigma_{2\mathrm{post}}^{2}}{n_{1}+n_{2}}\right)-\frac{1}{\sigma_{\mathrm{pre}}^{2}}\left(\frac{n_{2}\sigma_{1\mathrm{cov}}^{2}+n_{1}\sigma_{2\mathrm{cov}}^{2}}{n_{1}+n_{2}}\right)\right\},\;$ $\frac{{\left(\sigma_{1\mathrm{cov}}-\sigma_{2\mathrm{cov}}\right)}^{2}}{\left(n_{1}+n_{2}\right)\sigma_{\mathrm{pre}}^{2}},\;\frac{{\left(\sigma_{1\mathrm{cov}}-\sigma_{2\mathrm{cov}}\right)}^{2}}{\left(n_{1}+n_{2}\right)\sigma_{\mathrm{pre}}^{2}}$
*t*‐test_ChangeEV	$\left(\frac{1}{n_{1}}+\frac{1}{n_{2}}\right)\left\{\left(\frac{n_{1}\sigma_{1\mathrm{post}}^{2}+n_{2}\sigma_{2\mathrm{post}}^{2}}{n_{1}+n_{2}}\right)+\sigma_{\mathrm{pre}}^{2}-2\left(\frac{n_{1}\sigma_{1\mathrm{cov}}+n_{2}\sigma_{2\mathrm{cov}}}{n_{1}+n_{2}}\right)\right\},\;$ $\frac{\left(n_{1}-n_{2}\right)(\sigma_{1\mathrm{post}}^{2}-\sigma_{2\mathrm{post}}^{2})}{n_{1}n_{2}}-\frac{2\left(n_{1}-n_{2}\right)\left(\sigma_{1\mathrm{cov}}-\sigma_{2\mathrm{cov}}\right)}{n_{1}n_{2}},\;0$
*t*‐test_PostEV	$\left(\frac{1}{n_{1}}+\frac{1}{n_{2}}\right)\left(\frac{n_{1}\sigma_{1\mathrm{post}}^{2}+n_{2}\sigma_{2\mathrm{post}}^{2}}{n_{1}+n_{2}}\right),\;\frac{\left(n_{1}-n_{2}\right)(\sigma_{1\mathrm{post}}^{2}-\sigma_{2\mathrm{post}}^{2})}{n_{1}n_{2}},\;0$

a The bias is calculated by model‐based asymptotic variance minus asymptotic variance.

### 3.3 ANCOVA and *t*‐test and asymptotic properties

In this section, we show the asymptotic variances of the treatment effect estimators in four ANCOVAs and four *t*‐tests. The ordinary least squares (OLS) estimator of the treatment effect in the ANCOVA with equal slopes and equal residual variances, ANCOVA_ESEV, is a consistent estimator under random allocation and its asymptotic variance is the same as formula (II) or formula (VI) for $n_{1}=n_{2}$ in Table 2 (Funatogawa et al., 2011; Yang & Tsiatis, 2001). The asymptotic variance, model‐based asymptotic variance, and its bias are given in Funatogawa et al. (2011), and these are the same as those of EMEV.

Funatogawa et al. (2011) showed the properties of the ANCOVA with equal slopes and unequal residual variances, ANCOVA_ESUV, under random allocation. The generalized least squares (GLS) estimator of the treatment effect is obtained by ${\hat{\beta }}_{\mathrm{gls}}={\left(X^{\prime }V^{-1}X\right)}^{-1}\;X^{\prime }V^{-1}Y$. Here, $Y=(Y_{112},\ldots ,Y_{1n_{1}2},Y_{212},\ldots ,Y_{2n_{2}2})$ and $X=(X_{11},\ldots ,X_{1n_{1}},X_{21},\ldots ,X_{2n_{2}})$ with $\;\;X_{ij}=\left(\begin{array}{ccc} 1 & Y_{ij1} & x_{ij} \end{array}\right)$ and $\beta ={\left(\begin{array}{ccc} \beta_{\mathrm{int}} & \beta_{\mathrm{slope}} & \beta_{\mathrm{trt}} \end{array}\right)}^{\prime }$. **V** is an $\left(n_{1}+n_{2}\right)\times \left(n_{1}+n_{2}\right)$ diagonal matrix whose *l*‐th diagonal elements are the residual variances $\sigma_{1\mathrm{res}}^{2}\;\left(l=1,\ldots ,n_{1}\right)$ and $\sigma_{2\mathrm{res}}^{2}\;\left(l=n_{1}+1,\ldots ,n_{1}+n_{2}\right)$. The asymptotic variance of ${\hat{\beta }}_{\mathrm{trt}}$ is given as formula (III) or (VII) for $n_{1}=n_{2}$ in Table 2. Because $\sigma_{1\mathrm{res}}^{2}$ and $\sigma_{2\mathrm{res}}^{2}$ are unknown, we often estimate these variances using likelihood‐based methods under the assumption of normality. Consequently, this estimator is based on maximum likelihood methods.

In the ANCOVA with unequal slopes, the difference of expected values of posttreatment measurements between groups depends on pretreatment measurements, and the treatment effect is often estimated at the observed mean of pretreatment measurements. In the ANCOVA of unequal slopes with equal residual variances, ANCOVA_USEV, the OLS estimator of the treatment effect at the observed mean is a consistent estimator for the treatment effect at the true mean (Yang & Tsiatis, 2001). This OLS estimator is identical with the GLS estimator for the ANCOVA of unequal slopes with unequal residual variances, ANCOVA_USUV. The asymptotic variance of the treatment effect estimator is the same as formula (I) in Table 2 (Funatogawa & Funatogawa, 2011; Yang & Tsiatis, 2001). However, it differs from the model‐based asymptotic variances given in Table 3. In ANCOVA_USUV irrespective of equal or unequal sample sizes and ANCOVA_USEV under equal sample sizes, the bias is ${\left(\sigma_{1\mathrm{cov}}-\sigma_{2\mathrm{cov}}\right)}^{2}{\left\{\left(n_{1}+n_{2}\right)\sigma_{\mathrm{pre}}^{2}\right\}}^{-1}$, and the model‐based variances are always underestimated and the tests are liberal. This is caused because it is not corrected for estimating the unknown pretreatment expectation (Chen, 2006; Funatogawa & Funatogawa, 2011; Winkens et al., 2007). In ANCOVA_USEV under unequal sample sizes, there are both conservative and liberal cases.

The point estimate of the *t*‐test on change with unequal variances, *t*‐test_ChangeUV, is the same as that with equal variances, *t*‐test_ChangeEV. Under random allocation with $\sigma_{1\mathrm{pre}}^{2}=\sigma_{2\mathrm{pre}}^{2}$, it is given as formula (IV) or (VIII) for $n_{1}=n_{2}$ in Table 2. The point estimate of the *t*‐test on posttreatment measurements with unequal variances, *t*‐test_PostUV, is the same as that with equal variances, *t*‐test_PostEV, and the asymptotic variance of the treatment effect estimator is given as formula (V) or (IX) for $n_{1}=n_{2}$ in Table 2. The model‐based asymptotic variances are correct in *t*‐test_ChangeUV and *t*‐test_PostUV irrespective of equal or unequal sample sizes and *t*‐test_ChangeEV and *t*‐test_PostEV under equal sample sizes. However, these are incorrect in *t*‐test_ChangeEV and *t*‐test_PostEV under unequal sample sizes as given in Table 3.

### 3.4 Comparison of asymptotic variances

In this section, we compare the asymptotic variances of the treatment effect estimators. Let *V*_I_ to *V*_V_ be the asymptotic variances for formulae (I)–(V) shown in Table 2. We compare *V*_I_ with the other asymptotic variances. *V*_II_–*V*_I_ is

(1) $$\left(\frac{1}{n_{1}}+\frac{1}{n_{2}}\right)\frac{1}{\sigma_{\mathrm{pre}}^{2}}{\left(\frac{n_{1}-n_{2}}{n_{1}+n_{2}}\right)}^{2}{\left(\sigma_{1\mathrm{cov}}-\sigma_{2\mathrm{cov}}\right)}^{2}.$$

Therefore, $V_{I}\leq V_{\mathrm{II}}$, and equality holds under equal sample sizes. Although equality also holds under equal covariances, we consider data with unequal covariances in this paper. The formula (1) is the same as the difference of the asymptotic variances between the ANCOVA_ESEV and ANCOVA_USEV in Funatogawa and Funatogawa (2011). $V_{I}\leq V_{\mathrm{III}}$ from the difference of the asymptotic variances between the ANCOVA_ESUV and ANCOVA_USEV in Funatogawa and Funatogawa (2011). $V_{\mathrm{IV}}-V_{I}$ is

(2) $$\left(\frac{1}{n_{1}}+\frac{1}{n_{2}}\right)\frac{1}{\sigma_{\mathrm{pre}}^{2}}{\left(\sigma_{\mathrm{pre}}^{2}-\frac{n_{2}\sigma_{1\mathrm{cov}}+n_{1}\sigma_{2\mathrm{cov}}}{n_{1}+n_{2}}\right)}^{2}.$$

Therefore, $\;V_{I}\leq V_{\mathrm{IV}}$, and equality holds when the pretreatment variance equals the weighted mean of covariances. Under $n_{1}=n_{2}$, formula (2) is $2n^{-1}\sigma_{\mathrm{pre}}^{-2}{\left\{\sigma_{\mathrm{pre}}^{2}-\left(\sigma_{1\mathrm{cov}}+\sigma_{2\mathrm{cov}}\right)/2\right\}}^{2}$, and $V_{I}=V_{\mathrm{II}}\;\leq V_{\mathrm{IV}}$. Lastly, $V_{I}\leq V_{V}$.

## 4 SIMULATION STUDIES AND NUMERICAL EXAMPLE

### 4.1 Simulation studies

For continuous pre‐ and posttreatment measurements in randomized trials, we compared the following methods: three longitudinal models, four ANCOVAs, and four *t*‐tests. We compared actual type I error rates of these methods with a two‐sided nominal type I error rate of 5% and root relative mean squared errors (RRMSE) in simulated experiments. We simulated 100,000 data sets with unequal covariances and posttreatment variances by the following model:

$$\left(\begin{array}{c} Y_{ij1}\\ Y_{ij2} \end{array}\right)∼\mathrm{MVN}\left\{\left(\begin{array}{c} \mu_{\mathrm{pre}}\\ \mu_{i\;\mathrm{post}} \end{array}\right),\left(\begin{array}{cc} \sigma_{\mathrm{pre}}^{2} & \sigma_{i\;\mathrm{cov}}\\ \sigma_{i\;\mathrm{cov}} & \sigma_{i\;\mathrm{post}}^{2} \end{array}\right)\right\},$$

where $Y_{ijk}$ is, for the *i*‐th (i=1,2) treatment group, the measurement of the *j*‐th $\left(j=1,\ldots ,n_{i}\right)$ subject at the *k*‐th (k=1,2) time. MVN represents a multivariate normal distribution. If an actual type I error rate is 5%, the 95% confidence interval is 4.86–5.14% with 100,000 data sets.

We set the parameters of variance–covariance matrices as $(\sigma_{\mathrm{pre}}^{2},\;\sigma_{i\;\mathrm{cov}},\;\sigma_{i\;\mathrm{post}}^{2})\;$= (25,15,59) for Group 1 (i=1) and (25,23,30) for Group 2 (i=2). For the parameter setting, we refer to the estimates of the blood lead data introduced in Section 2, as well as Funatogawa et al. (2011) and Funatogawa and Funatogawa (2011). Note that the means and variances of pretreatment measurements are equal between groups. In this parameter setting, the residual variances of Group 1 and Group 2 are about 50 and 10 in the ANCOVA, 54 and 9 in the *t*‐test on change and 59 and 30 in the *t*‐test on posttreatment measurements. In each method, Group 1 has a larger variance. The slopes in the ANCOVA are 15/25 = 0.6 and 23/25≈0.9, respectively, and the correlation coefficients between pre‐ and posttreatment measurements are $15/(\sqrt{25}\sqrt{59})\approx 0.4$ and $23/(\sqrt{25}\sqrt{30})\approx 0.8,$ respectively.

The sample sizes in each group are set to $\left(n_{1},\;n_{2}\right)$ = (300, 300), (400, 200), and (200, 400) as large sample‐size situations. In the first case, sample sizes are equal, in the second case, a group with a large sample size has a larger variance $\left(n_{1}>n_{2}\right)$, and in the last case, a group with a larger sample size has a smaller variance $\left(n_{1}<n_{2}\right)$. Similarly, the numbers are also set to $\left(n_{1},\;n_{2}\right)$ = (45, 45), (60, 30), and (30, 60) as moderate sample‐size situations. We use the Kenward–Roger approximation (Kenward & Roger, 1997) for longitudinal models and the Satterthwaite approximation for the degrees of freedom (Satterthwaite, 1946) for the ANCOVAs and *t*‐tests. To obtain actual type I error rates, we calculate the proportions of data sets in which a significant difference was detected under $\mu_{1\mathrm{post}}=\mu_{2\mathrm{post}}$. Note that the proportions for which the true treatment effect is included in the 95% confidence intervals (coverage proportion) are expressed by subtracting the actual type I error rates (%) from 100% under $\mu_{1\mathrm{post}}\neq \mu_{2\mathrm{post}}$. In the simulation studies and analysis of numerical examples in the next section, we use the SAS 9.4, SAS proc MIXED for the longitudinal models and ANCOVAs, SAS proc ttest for the *t*‐tests. The program codes of the longitudinal models are provided in the Appendix. The program codes of the ANCOVAs are provided in Funatogawa and Funatogawa (2011). Source code to reproduce the results is available as Supporting Information on the journal's web page (http://onlinelibrary.wiley.com/doi/10.1002/bimj.201800389/suppinfo).

The results are shown in Table 4. In large sample sizes, the actual type I error rates of the two longitudinal models with unequal variances (EMVUV and EMUV) were close to the nominal level, even in unequal sample sizes. The ANCOVA with equal slopes and unequal residual variances (ANCOVA_ESUV), and the *t*‐tests with unequal variances were also close to the nominal level. The actual type I error rates of the longitudinal models with equal variance matrices (EMEV), ANCOVA_ESEV, and the *t*‐tests with equal variances were close to the nominal level only in equal sample sizes. The ANCOVAs with unequal slopes (ANCOVA_USs: ANCOVA_USUV and ANCOVA_USEV) did not keep a nominal type I error rate even in equal sample sizes. The actual type I error rates of the methods with equal variances (EMEV, ANCOVA_ESEV, ANCOVA_USEV, and *t*‐tests with equal variances) were less than 5%, that is conservative, when the group with a larger variance had a larger sample size ($n_{1}>n_{2}$), and these were more than 5%, that is liberal, when the group with a larger variance had a smaller sample size ($n_{1}<n_{2}$).

**Table 4:** Actual type I error rates and RRMSEs in the simulation studies for the data with unequal covariances and unequal variances of posttreatment measurements under random allocation with large and moderate sample sizes

		Large sample sizes^a^			Moderate sample sizes^a^		
		300:300	400:200	200:400	45:45	60:30	30:60
Type I error rate (%)							
Longitudinal	EMVUV EMUV EMEV	4.95 4.95 4.97	4.89 4.87 1.36	4.98 4.99 12.20	5.04 5.02 5.18	5.02 5.02 1.51	5.11 5.11 12.42
ANCOVA	USUV USEV ESUV ESEV	5.20 5.23 4.96 4.97	5.16 1.38 4.87 1.36	5.13 12.57 4.95 12.20	5.28 5.44 5.01 5.16	5.33 1.43 5.02 1.51	5.24 13.00 5.02 12.39
*t*‐test	ChangeUV ChangeEV PostUV PostEV	5.01 5.03 5.02 5.02	4.89 1.22 4.96 2.84	4.94 12.39 4.98 7.89	5.04 5.19 5.07 5.08	4.97 1.36 4.96 2.91	4.99 12.71 5.01 7.92
RRMSE							
Longitudinal	EMVUV EMUV EMEV	4.07 4.07 4.07	3.78 3.78 3.80	4.78 4.78 4.79	10.6 10.6 10.6	9.8 9.8 9.9	12.5 12.5 12.4
ANCOVA	US^b^ ESUV ESEV	4.07 4.09 4.07	3.78 3.78 3.80	4.78 4.83 4.79	10.6 10.6 10.6	9.8 9.8 9.9	12.5 12.5 12.4
*t*‐test	Change^b^ Post^b^	4.16 4.96	3.84 4.96	4.91 5.53	10.8 12.8	10.0 12.8	12.7 14.3

a Sample size of the group with a larger variance: sample size of the group with a smaller variance.

b The estimates with equal and unequal variances are identical for ANCOVAs with unequal slopes and *t*‐tests.

The RRMSEs of EMVUV and ANCOVA_USs (ANCOVA_USUV and ANCOVA_USEV) were the smallest in large sample sizes irrespective of allocation ratios. However, ANCOVA_USs did not keep a nominal type I error rate. The RRMSEs of EMUV, which has redundant unequal baseline variances, were the second smallest. In equal sample sizes, the RRMSEs of EMEV and ANCOVA_ESEV were also the second smallest. The RRMSEs of the best and second‐best models were similar, and the differences were too small to be detected in Table 4. The ANCOVA with equal slopes and unequal residual variances (ANCOVA_ESUV) was less efficient compared to EMVUV, EMUV, and ANCOVA_USs, but the loss of efficiency was small compared to the *t*‐tests on change. The *t*‐test on posttreatment measurements was not efficient.

In moderate sample sizes, the results were similar with those of large sample sizes. Under $\left(n_{1},\;n_{2}\right)=\left(30,60\right)$, the actual type I error rates were 5.11 for EMVUV and EMUV, and slightly larger than the nominal level of 5%. Under $\left(n_{1},\;n_{2}\right)=\left(45,45\right)$, the actual type I error rates were slightly larger than the nominal level for the methods with equal variances (EMEV, ANCOVA_ESEV, *t*‐test_ChangeEV, and *t*‐test_PostEV). Under $\left(n_{1},\;n_{2}\right)=\left(30,60\right)$, EMEV and ANCOVA_ESEV showed slightly better efficiency than EMVUV, EMUV, and ANCOVA_USs, but these models with equal variances did not keep a nominal type I error rate.

### 4.2 Numerical example

We applied the statistical methods to the numerical examples shown in Section 2, and the results are shown in Table 5. The sample sizes of the active and placebo groups are *n*_1_ and *n*_2_, respectively, and the posttreatment variance of the active group is larger than that of the placebo group. When $n_{1}=n_{2}=50$, the treatment effect estimates of EMVUV, EMUV, EMEV, ANCOVA_USs, and ANCOVA_ESEV were almost the same, and the standard errors (SEs) were also similar.

**Table 5:** Estimates of the treatment effect in numerical example

		Equal sample sizes^a^		Unequal sample sizes^a^		Unequal sample sizes^a^	
		50:50		50:25		25:50	
		Estimate	SE	Estimate	SE	Estimate	SE
Longitudinal	EMVUV EMUV EMEV	11.23 11.23 11.23	1.13 1.13 1.13	10.26 10.25 10.39	1.25 1.25 1.55	11.45 11.44 11.43	1.34 1.34 1.10
ANCOVA	USUV USEV ESUV ESEV	11.23 * 11.24 11.23	1.11 1.11 1.13 1.12	10.26 * 10.24 10.39	1.23 1.53 1.25 1.54	11.45 * 11.42 11.43	1.31 1.07 1.34 1.09
*t*‐test	ChangeUV ChangeEV PostUV PostEV	11.26 ** 11.13 ***	1.15 ** 1.34 ***	10.11 ** 11.09 ***	1.25 1.57 1.66 1.78	11.41 ** 11.51 ***	1.37 1.11 1.50 1.43

a Sample size of the active group with a larger variance: sample size of the placebo group with a smaller variance.

^*^The estimates with equal and unequal variances are identical for ANCOVAs with unequal slopes.

^**^The values are identical with those of *t*‐test_ChangeUV.

^***^The values are identical with those of *t*‐test_PostUV.

We then examined the influences of unequal sample sizes reducing half of data from one group; $\left(n_{1},n_{2}\right)=\left(50,\;25\right)$ as the case a group with a large sample size has a larger variance and $\left(n_{1},n_{2}\right)=\left(25,\;50\right)$ as the case a group with a large sample size has a smaller variance. These data are shown in Figure 1. The SEs of the methods with unequal variances (EMVUV, EMUV, ANCOVA_USUV, ANCOVA_ESUV, and *t*‐test_ChangeUV) under $\left(n_{1},n_{2}\right)=\left(50,\;25\right)$ were smaller than those under $\left(n_{1},n_{2}\right)=\left(25,\;50\right)$. These SEs under reduced sample sizes were larger than those under equal sample sizes, $\left(n_{1},n_{2}\right)=\left(50,\;50\right)$. In contrary, the SEs of the methods with equal variances (EMEV, ANCOVA_USEV, ANCOVA_ESEV, and *t*‐test_ChangeEV) under $\left(n_{1},n_{2}\right)=\left(50,\;25\right)$ were larger than those under $\left(n_{1},n_{2}\right)=\left(25,\;50\right)$ and the SEs of the above methods with unequal variances under $\left(n_{1},n_{2}\right)=\left(50,\;25\right)$. The SEs of the methods with equal variances under $\left(n_{1},n_{2}\right)=\left(25,\;50\right)$ were even smaller than those under the equal sample sizes $\left(n_{1},n_{2}\right)=\left(50,\;50\right)$, although the former data are the reduced sample from the latter data. These results were caused by the wrong assumption of equal variances, and these models were conservative under $n_{1}>n_{2}$ and liberal under $n_{1}<n_{2}$. These results of these numerical examples correspond to those of the simulation studies. Because the *t*‐test on posttreatment measurements does not take into account the baseline measurements and the ratio of posttreatment variances was not relatively large in this example, the results of this method differed from those of the other methods.

## 5 DISCUSSION

Longitudinal models with equal baseline means and unequal covariances and posttreatment variances (EMVUV and EMUV) asymptotically keep a type I error rate and these are efficient in randomized trial. The assumption of equal baseline variances is not important for efficiency. Statistical models asymptotically do not keep nominal type I error rates if the model‐based asymptotic variance differs from the asymptotic variance, and these models are shown in Table 3 including ANCOVAs and *t*‐tests. The discrepancies occur by two reasons: the equal variance assumption and unequal slope assumption. Under equal sample sizes, the discrepancies caused by the equal variance assumption disappear, but the discrepancies caused by equal slope assumption still exist. The longitudinal model with equal variance matrices (EMEV) asymptotically keeps a type I error rate and its efficiency is the same as EMVUV and EMUV under equal sample sizes, but it does not keep a type I error rate under unequal sample sizes.

Funatogawa and Funatogawa (2011) provided the details of the order of the asymptotic efficiency for the treatment effect estimators among ANCOVAs. Based on Tables 2 and 3, EMVUV and EMUV correspond to ANCOVAs with unequal slopes (ANCOVA_USUV and ANCOVA_USEV) regarding only the point estimate of the treatment effect. ANCOVA_USUV and ANCOVA_USEV have the same high efficiency as EMVUV and EMUV, but asymptotically do not keep a type I error rate. EMEV corresponds to the ANCOVA with equal slopes and equal residual variances (ANCOVA_ESEV) regarding the point estimate and the model‐based variances. EMEV and ANCOVA_ESEV have the same characteristics. The ANCOVA with equal slopes and unequal residual variances (ANCOVA_ESUV) asymptotically keeps a type I error rate and the loss of efficiency is small under either equal or unequal sample sizes. When there are missing data, the longitudinal models use all observed data, but the ANCOVAs and *t*‐tests on change use only paired data.

Unequal allocation of patients to treatments is sometimes applied in clinical trials. The following properties are known for the *t*‐test with equal variances, the Student's *t*‐test (Algina, 2005). In unequal sample sizes and unequal variances, the actual type I error rate is not at a nominal. The actual rate is over a nominal level when a group with a large sample size has a smaller variance, and that is under a nominal level when a group with a large sample size has a larger variance. These properties are applied to the ANCOVAs with equal residual variances (Funatogawa & Funatogawa, 2011; Funatogawa et al., 2011) and the longitudinal model with equal variance matrices. Because large discrepancies from the nominal rate can occur, the longitudinal model with equal variance matrices should not be used when the sample sizes and covariances and variances of posttreatment measurements are unequal between groups.

Funatogawa et al. (2011) discussed a conceptual data‐generating model under random allocation with two random subject effects for the true pre‐ and posttreatment measurements, respectively. The number of the variance parameters in this model is too large to estimate from the pre‐ and posttreatment measurements data. Crager (1987) considered a model assuming a common random subject effect for the true pre‐ and posttreatment measurements. The variance–covariance matrices are equal between two groups, and the model corresponds to EMEV in this paper.

In this paper, we examined longitudinal models with equal baseline means because of random allocation. Under random allocation, the longitudinal model with unequal baseline means and unequal variance–covariance matrices has redundant parameters. Let $\mu_{i\;\mathrm{pre}}$ be the pretreatment mean of the *i*‐th treatment group. The treatment effect is estimated by $\left(\mu_{2\mathrm{post}}-\mu_{2\mathrm{pre}}\right)-\left(\mu_{1\mathrm{post}}-\mu_{1\mathrm{pre}}\right)$ based on this model. It corresponds to the *t*‐test on change with unequal variances regarding the point estimate and the model‐based variance, and those are identical if there are no missing data. The asymptotic variance is

$$\left(\frac{1}{n_{1}}+\frac{1}{n_{2}}\right)\left\{\left(\frac{n_{2}\sigma_{1\mathrm{post}}^{2}+n_{1}\sigma_{2\mathrm{post}}^{2}}{n_{1}+n_{2}}\right)+\left(\frac{n_{2}\sigma_{1\mathrm{pre}}^{2}+n_{1}\sigma_{2\mathrm{pre}}^{2}}{n_{1}+n_{2}}\right)-2\left(\frac{n_{2}\sigma_{1\mathrm{cov}}+n_{1}\sigma_{2\mathrm{cov}}}{n_{1}+n_{2}}\right)\right\}.$$

Under random allocation with $\sigma_{1\mathrm{pre}}^{2}=\sigma_{2\mathrm{pre}}^{2}$, the asymptotic variance is given as formula (IV) for arbitrary allocation ratios or formula (VIII) for $n_{1}=n_{2}$ in Table 2. The treatment effect is also estimated by $\mu_{2\mathrm{post}}-\mu_{1\mathrm{post}}$ based on this model. It corresponds to the *t*‐test on posttreatment measurements with unequal variances, and those are identical if there are no missing data. These are less efficient. The assumption of equal baseline means is important for efficiency.

## CONFLICT OF INTEREST

The authors have declared no conflict of interest.

## Supporting information

Supporting Information

## APPENDIX 1

### A.1 Program codes

We provide the SAS codes of the MIXED procedure to obtain estimates of the longitudinal models of EMVUV and EMUV in Section 3.2. For EMEV, just remove the GROUP = trt option from the REPEATED statement of EMUV. In the following code, y is pre‐ and posttreatment measurements, no is an indicator of subjects, trt is a continuous variable for the groups taking 0 or 1, t is a continuous variable for the time taking 0 at the pretreatment and 1 at the posttreatment. Time1 is a discrete variable and takes two levels, such as time1 = t. Time2 is a discrete variable and takes three levels. For example, time2 = 0 if t = 0, time2 = 1 if t = 1 and trt = 0, and time2 = 2 if t = 1 and trt = 1.

/* Longitudinal EMVUV: equal baseline means and variances and unequal covariances and posttreatment variances */

PROC MIXED METHOD = REML;

  CLASS no time2;

  MODEL y = t trt*t/ DDFM = KR;

  REPEATED time2/ TYPE = UN SUBJECT = no;

  ESTIMATE ‘diff’ trt*t ‐1;

RUN;

/* Longitudinal EMUV: equal baseline means and unequal variance covariance matrices */

PROC MIXED METHOD = REML;

  CLASS no time1;

  MODEL y = t trt*t/ DDFM = KR;

  REPEATED time1/ TYPE = UN SUBJECT = no GROUP = trt;

  ESTIMATE ‘diff’ trt*t ‐1;

RUN;
